# Spatial and temporal trends of dengue infections in Curaçao: A 21-year analysis

**DOI:** 10.1016/j.parepi.2024.e00338

**Published:** 2024-01-26

**Authors:** Bart Roelofs, Daniella Vos, Yaskara Halabi, Izzy Gerstenbluth, Ashley Duits, Maria E. Grillet, Adriana Tami, Maria F. Vincenti-Gonzalez

**Affiliations:** a University of Groningen, Faculty of Spatial Sciences, Groningen, the Netherlands; b GGD Curaçao, Ministry of Health, Curaçao; c Red Cross Blood Bank Foundation Curaçao, Curaçao; d Laboratorio de Biología de Vectores y Parásitos, Instituto de Zoología y Ecología Tropical, Facultad de Ciencias, Universidad Central de Venezuela, Caracas, Venezuela; e University of Groningen, University Medical Center Groningen, Department of Medical Microbiology and Infection Prevention, Groningen, the Netherlands

**Keywords:** Dengue fever, Epidemiology, Vector-borne disease, Infectious disease control, Spatio-temporal analysis

## Abstract

Dengue viruses are a significant global health concern, causing millions of infections annually and putting approximately half of the world's population at risk, as reported by the World Health Organization (WHO). Understanding the spatial and temporal patterns of dengue virus spread is crucial for effective prevention of future outbreaks. By investigating these patterns, targeted dengue surveillance and control measures can be improved, aiding in the management of outbreaks in dengue-affected regions. Curaçao, where dengue is endemic, has experienced frequent outbreaks over the past 25 years. To examine the spatial and temporal trends of dengue outbreaks in Curaçao, this study employs an interdisciplinary and multi-method approach. Data on >6500 cases of dengue infections in Curaçao between the years 1995 and 2016 were used. Temporal and spatial statistics were applied. The Moran's I index identified the presence of spatial autocorrelation for incident locations, allowing us to reject the null hypothesis of spatial randomness. The majority of cases were recorded in highly populated areas and a relationship was observed between population density and dengue cases. Temporal analysis demonstrated that cases mostly occurred from October to January, during the rainy season. Lower average temperatures, higher precipitation and a lower sea surface temperature appear to be related to an increase in dengue cases. This effect has a direct link to La Niña episodes, which is the cooling phase of El Niño Southern Oscillation. The spatial and temporal analyses conducted in this study are fundamental to understanding the timing and locations of outbreaks, and ultimately improving dengue outbreak management.

## 1 Introduction

Dengue is a major public health concern, causing millions of infections annually. In recent years, the Caribbean region experienced multiple epidemic outbreaks (Pan American Health Organization, 2023). Island habitats are heavily affected by dengue, and the relationship between climatic variables and dengue virus occurrence needs to be studied more profoundly (Lowe et al., 2018). In addition to this, the Caribbean region has experienced an unprecedented crisis of co-occurring epidemics of febrile illness, such as Chikungunya and Zika viruses (Lowe et al., 2020). A recent study by Kim et al. (Kim et al., 2023) found that a dengue virus infection can impact subsequent Zika infections, adding an extra layer of importance to the study of dengue virus infections. The dengue virus, similarly to Chikungunya and Zika, is transmitted to humans by the *Aedes Aegypti* mosquito.

To understand and investigate the development of dengue virus infections, it is important to consider a combination of spatial patterns and temporal trends. Therefore, investigation of spatial heterogeneity in combination with ecological processes is key to understanding insect populations (Vinatier et al., 2011). Spatial trends of Vector Borne Diseases (VBD) have been studied extensively, and earlier studies have already portrayed the importance of spatial analysis of incidence data (Churakov et al., 2019; Freitas et al., 2019; Akter et al., 2019). However, exhaustive and focal (point-pattern based) spatial-temporal approaches are scarce for studies of VBD.

Climatic trends are another key aspect found to affect dengue virus occurrence. There is, however, conflicting evidence on the direction of this relationship. On the one hand, there are multiple studies reporting a relationship between an increase in temperature and dengue cases, such as in Venezuela (Vincenti-Gonzalez et al., 2018) Brazil (Bavia et al., 2020), Puerto Rico (Jury, 2008), and Barbados (Lowe et al., 2018). On the other hand, studies in Curaçao (Limper et al., 2010; Limper et al., 2016) and Guadeloupe (Gharbi et al., 2011) identified a decrease in mean temperature to be related to more dengue cases. Relevant to this aspect is the occurrence of El Niño Southern Oscillation (ENSO), as there are multiple studies (Vincenti-Gonzalez et al., 2018; Xiao et al., 2017; Pramanik et al., 2020) that revealed a link between dengue and the warming phase of ENSO.

Whether due to changing climate conditions, vector or human movement, or other factors, dengue has been spreading to formerly unaffected areas (Kraemer et al., 2019). It is therefore of importance to conduct research that aims to understand the underlying spatial and temporal mechanisms related to dengue spread.

Curaçao is an island in the Caribbean that experienced multiple dengue outbreaks over the past decades. This paper extensively explores the spatial and temporal aspects of dengue incident data, with the twofold aim of increasing our understanding of the spatial and temporal trends of dengue to inform about suitable outbreak management in Curaçao and elsewhere, and testing the efficacy of a combined spatial and temporal approach in disentangling underlying mechanisms of dengue spread. This was done by conducting spatial and temporal analysis on dengue incident data and climatic variables from the period 1995–2016 on the island of Curaçao.

It is crucial to generate and analyze a combination of different sources of data, such as epidemiological data, spatial data, and climatic data, to anticipate future epidemics. Areas at risk for dengue outbreaks can benefit from spatial and temporal dengue research, for example by improving targeted dengue surveillance and control, as well as better funding allocation. This might be the route towards an effective outbreak management in regions suffering from dengue.

## 2 Methods

### 2.1 Study area and epidemiological data

**Fig. 1:**
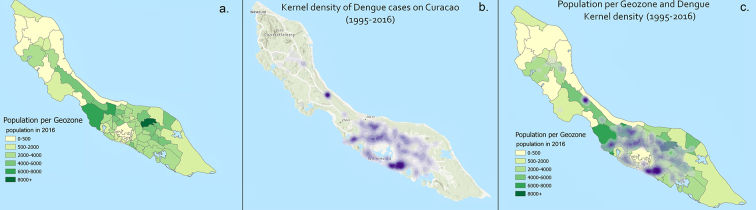
a) Population distribution per geozone b) Kernel density of dengue cases and c) Population distribution and density of dengue cases. Basemap: ESRI.

The island of Curaçao is located in the southern Caribbean Sea, 37 miles (60 km) north of the coast of Venezuela. Curaçao has a hot, semi-arid climate with a dry season from January to September and a wet season from October to December (Meteo Curaçao, 2020). The capital is Willemstad, and the vast majority of the roughly 160,000 inhabitants live in concentrated areas towards the east side of the island and in the capital (136,660 pop) (Fig. 1).

The epidemiological data covers 6913 registered dengue infections in Curaçao over a 21-year period (1995–2016) and was provided by the Ministry of Health (MoH) of Curaçao. The data was analyzed anonymously, and individuals were coded with unique numeric identifiers. The dengue case data was obtained in tabular format, containing patient ID and home address. Monthly measures of temperature and precipitation were obtained from the Hato Airport Meteorological weather station. We used monthly sea surface temperatures (SST) of the eastern and central tropical Pacific as an index of ENSO-region 3.4 (Niño 3.4 index). The SST time-series were obtained from the Climate Prediction Center of the National Oceanic and Atmospheric Administration (Climate prediction Centre (CPC), n.d.).

### 2.2 Geocoding process

The latitude and longitude information was obtained by geocoding the address information provided in the dengue cases dataset. For this, the Local Focus geocoder (Local focus geocoder, 2020) was used, which makes use of Open Street map data (©OpenStreetMap), as well as the Pelias geocoder (github.com/pelias/pelias). Since the geocoded data is represented on a household level, no maps displaying individual data points were included in this manuscript to prevent patients being identifiable based on their home addresses. Of the 6913 cases in total, 4.9% (*n* = 341) were not possible to geocode, due to duplicates or insufficient address information.

### 2.3 Spatial analysis of dengue cases

Various analyses were conducted to gain insight on the spatial dynamics of dengue cases in Curaçao. First, we mapped the population density and dengue cases, and calculated kernel densities. The mean and median centers were calculated using the GPS case locations for every year, to study the change of the geographical center of the annual occurrences of dengue over the study period. The mean center is the geographical center of the yearly cases, which is calculated by taking the geographical mean of all equally weighted GPS locations. The median center, which is less affected by geographical outliers, is calculated by finding the location from the yearly GPS case locations that minimizes the overall Euclidean distance to all other case locations (Pimpler, 2017). Both the mean center and median center were calculated using ArcGIS Pro 2.5.1 (Environmental Systems Research Institute (ESRI), 2020).

Global Moran's I was used to test the spatial autocorrelation (Null hypothesis: observations are randomly distributed) among the total dataset features. Global Moran's I evaluates the spatial relationship of a set of features and associated attributes, providing a measure of spatial association in these features across the study area (Pfeiffer et al., 2008).By specifying a conceptual zone of a specific distance around each case, the influence of neighboring features can be simulated. In this analysis, both 150 m and 500 m were used as conceptual distances. The 150 m distance was selected to represent the maximum flight range of the *Aedes Aegypti* mosquito, while the 500 m distance represents larger-scale factors that can influence dengue spread, such as urbanization. The ‘zone of indifference’ setting was used to conceptualize the spatial relationship of our data, implying that neighboring features within a specific distance get assigned a higher weight which diminishes over distance outside of this range.

In addition to Global Moran's I, the Kulldorff's Scan was conducted to detect yearly space-time clusters (Kulldorff, 1997), using the SatScan software (BioMedWare, 2020). Kulldorff's Scan identifies the most likely clusters of dengue cases over space and time, using a circular scanning method. Within this method, a window of analysis containing 50% of total population and 50% of study period span was used.

Finally, optimized Hot spot analysis was conducted using the Getis-Ord Gi* statistic to identify hot and cold spots of dengue incidence (per 100,000 pop) using ArcGIS Pro 2.5.1. In these analyses, the cell size was set to 150 m, based on the average and maximum flight range of the *Aedes aegypti* mosquito (Verdonschot and Besse-Lototskaya, 2014).

### 2.4 Temporal analysis of dengue cases

A time series analysis was conducted on dengue cases by month using the statistical software R (R Core Team, 2020). The decomposition of these time series allowed for the comparison of trends and seasonality. The time series were further investigated using cross correlation functions, which calculated correlation effects between the time series of climatic variables and dengue cases. The analysis shed a retrospective vision on the associations between climatic variables and dengue cases. Furthermore, the standardized anomalies of dengue incidence and Sea Surface Temperature (SST) were calculated by subtracting from each monthly observation the long-term (21 years) mean value of each particular month and dividing this by the long-term standard deviation, to explore whether there was a potential relationship between SST and dengue. These data were plotted against each other to display the different trends of these variables over the study period as compared to their baseline. Anomalies of more than +0.5 and − 0.5 are considered El Niño Southern Oscillation (ENSO) events (Anyamba et al., 2019). La Niña can be classified in the SST anomaly index as weak (0.5 to 0.9 SST anomaly), moderate (1.0 to 1.4 SST anomaly) or strong (>1.5 SST anomaly) (Vincenti-Gonzalez et al., 2018).

Epidemiological events and climatic time-series are non-stationary, meaning that they vary over time. To account for this, we used a specialized time series analysis method known as wavelet analyses (WA) to detect the periodic cycles and dominant components (*i.e.*, the most frequently repeated signal) of the time series and how they change over time. By representing the power of the time series as a function of the time and the duration period, the data was decomposed, which allowed us to gain insights into patterns over short as well as long durations of time (Schulte, 2016). Additionally, wavelet coherence (WC) methodology was used to compare the frequency components of dengue and climate time-series in order to quantify the statistical (linear) association between variables in a time span. Data were normalized before the analyses. The WA and WC analyses were performed using Matlab 2020a (MATLAB, 2019) and the toolboxes developed by Cazelles et al. (Cazelles et al., 2005).

## 3 Results

### 3.1 Spatial analysis

Fig. 1a shows the population distribution in Curaçao. The majority of the population lives in the southeastern part of the island, or in villages near the northwest. The distribution of dengue cases, displayed in density for privacy purposes, is visible in Fig. 1b with most cases clustered around Willemstad, the capital of Curaçao. In Fig. 1c, the case density is displayed overlaying the population map.

**Fig. 2:**
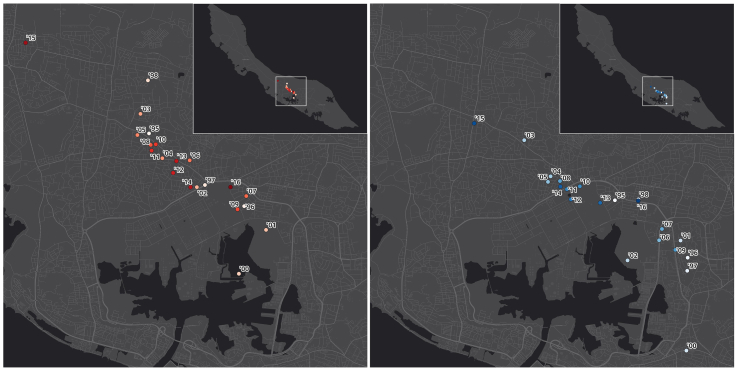
Mean (red) and Median (blue) centers, the number and intensity of the color corresponds to the year of epidemic (e.g.: ‘98 = 1998, ‘00 = 2000, ‘16 = 2016). Basemap: ESRI.

Fig. 2 shows the annual change in the center of each dengue outbreak, as determined by the mean and median center locations over time. The mean center is the mean location of all cases in a particular year in the dataset. Similarly, the median center is the median location of all cases in a specific year in the dataset. The spatial distribution pattern of the mean/median center for dengue cases shows clustering in these, as they move across the northwest, which is the most populated side of the island.

The total set of acquired cases were tested for spatial autocorrelation (the presence of systematic spatial variation in a variable) using Moran's I on two different distance bands. The first distance band, set to 150 m to represent the maximum *Aedes Aegypti* flight range, resulted in a Moran's Index of 0.06 and a *p*-value <0.0001. The second distance band, set to 500 m to represent a wider range of factors influencing dengue, resulted in a Moran's Index of 0.26 and a *p*-value <0.0001. These results confirm spatial autocorrelation in the dataset. The same analysis was conducted for the individual years and produced similar significant spatial autocorrelation results.

The Kulldorff's scan statistic was conducted for all years using dengue incidence on a monthly basis, to gain a first overview of dengue clustering. Statistically significant clusters were detected in every analyzed year. This method provided a most likely cluster (MLC), a second most likely cluster and a third most likely cluster. The location of the MLC shifted throughout the different years studied. Using a wide window of analysis allowed us to identify broad patterns, but this may have influenced the size of the MLC in certain years. The Getis-Ord Gi* hot spot analysis was therefore used to address and identify clustering at a local scale. The Kulldorff's scan results for each year are displayed in appendix 1.

**Fig. 3:**
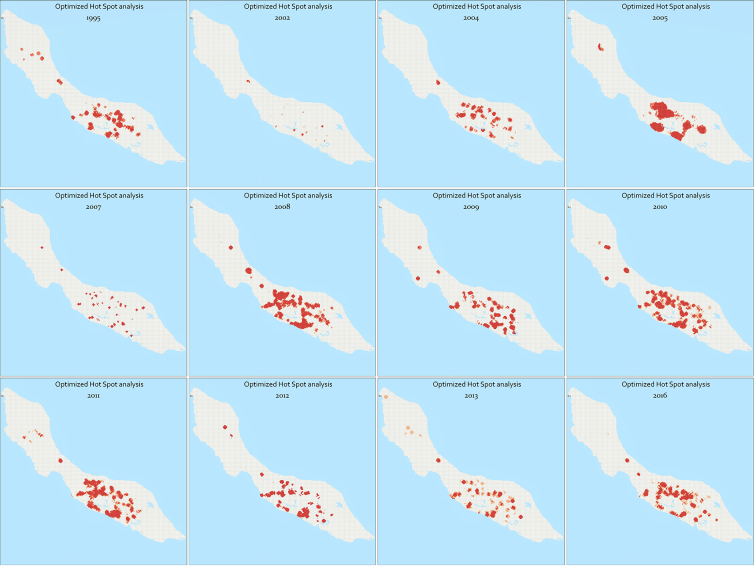
Hot Spots of dengue infections for years with *n* > 100 cases. Basemap: ESRI.

A more detailed cluster analysis was conducted using hot spot analysis, making use of the Getis-Ord Gi* statistic. Hot spot analysis was conducted for all study years where there were >100 cases per year (*n* > 100 cases). The analyses resulted in statistically significant hot spots for each tested year, which are displayed in Fig. 3. This figure highlights the areas with a notably high incidence of dengue cases for years recording over 100 cases. These hotspots were determined using the Local Getis-Ord Gi* statistic, indicating regions where Gi*(d) > 2.79 with a statistical significance of *p* < 0.05. Although the distribution of dengue cases per study year is highly heterogenous, the locations of hot spots appear to be clustered around the geozones close to Schottegat, the bay in the center of the island, and nearby areas. As is visible in Fig. 1, this area corresponds to a densely populated area. In addition, there were some persistent hot spots in the western part of the island, in the neighborhoods of Tera Cora and Barber.

### 3.2 Temporal analysis

**Fig. 4:**
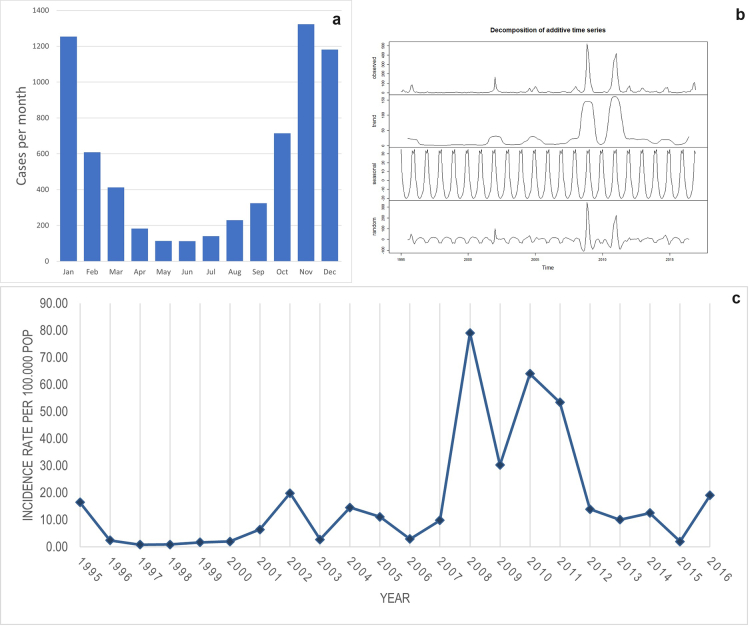
a) Temporal distribution of dengue cases by month over the total study period b) Temporal decomposition of dengue incidence in Curaçao, 1995–2016 c) Dengue Incidence rate (per 100,000 pop) per year.

During the wet season (October–February), the frequency of dengue cases was far greater compared to the dry season across all years (Fig. 4a). Fig. 4b shows the decomposition plot of the time series. In this figure, the observed cases (upper panel), the trend (second panel), the seasonality (third panel), and the randomness (fourth panel) are displayed. A strong seasonality in dengue case occurrence can be observed in the third panel. The cumulative incidence of dengue cases per 100,000 inhabitants per year ranged between 0.79 and 79.06 (median: 10.55; average: 17.06) (Fig. 4c). The decomposition of the time series of average temperature and precipitation are displayed in appendix 2.

**Fig. 5:**
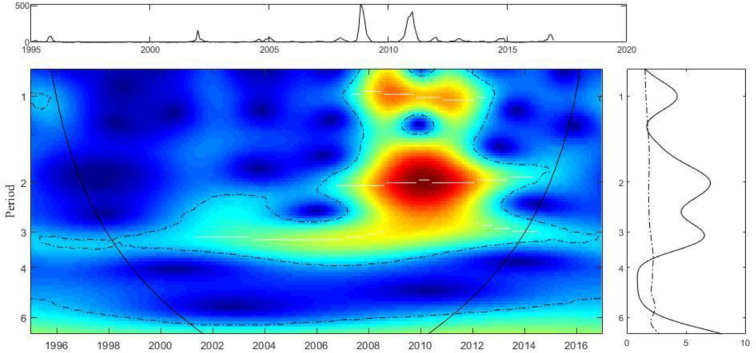
Wavelet power spectrum (WPS) of dengue cases. Main central panel: wavelet power spectrums (WPS) of dengue incidence. Right panel: Global spectrum (GS). Top panel over WPS: Original time-series of dengue incidence data. The y-axis of the WPS and GS describe the periods in years (e.g., period 1: annual cycles; period 2,3,4: inter-annual cycles). The x-axis of the GS shows the power at a given frequency (continuous line) with a significant threshold value of 5% (dashed line). In the WPS, the color code for power values ranges from dark blue for low values, to dark red for high ones. The areas surrounded by dotted-dashed lines are those including significant results (*p* < 0.05). The area within the cone of influence (continuous line) in the WPS indicates the region not influenced by edge effects.

The wavelet power spectrum (WPS) of dengue cases is displayed in Fig. 5. The main panel is the WPS, in which the y-axis represents the period in year cycles. In other words, period 1 indicates a 1-year cycle, period 2 indicates a 2- year cycle and so on. The x-axis represents the actual year of study. The color intensity indicates the power, with the spectrum ranging from dark blue for no power, through green and yellow to red for maximum power. The dashed lines indicate significant periods, and the curved line near the sides of the figure indicate parts of the data that are not affected by edge effects. The top panel displays the original time series of dengue cases. The right panel displays the Global Spectrum (GS), with the x-axis representing the power at a certain period-time range and the dashed line indicating the significant interval. This figure illustrates that dengue is significant on a 1-year cycle between 2007 and 2013, which implies a direct correspondence with seasonality. There is a strong significant 2-year cycle in the same period, and a weaker but longer trend on the three-year cycle.

### 3.3 Dengue, climate and ENSO

**Fig. 6:**
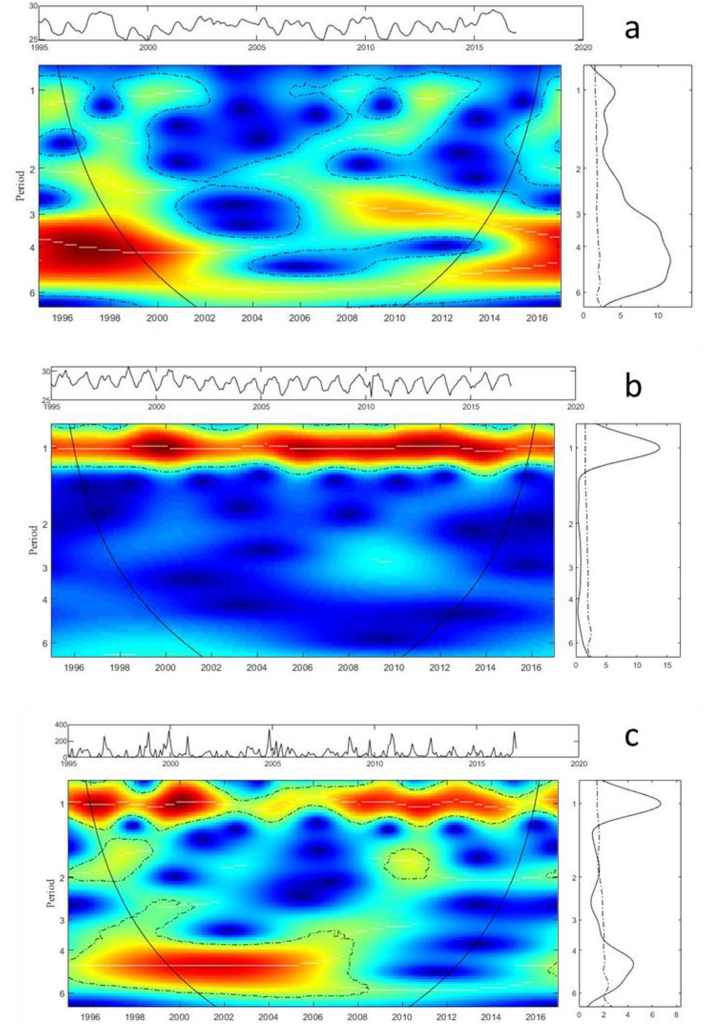
Patterns of interannual variability of the (a) SST, (b) average temperature and (c) precipitation. Main central panels: wavelet power spectrums (WPS) of SST, average temperature and precipitation. Right panels: Global spectrum (GS). Top panel over each WPS: Original time-series of the corresponding variable: SST, average temperature and precipitation. The y-axis of the WPS and GS describe the periods in years (e.g., period 1: annual cycles; period 2,3,4: interannual cycles). The x-axis of the GS shows the power at a given frequency (continuous line) with its significant threshold value of 5% (dashed line). In the WPS, the color code for power values ranges from dark blue for low values, to dark red for high ones. The areas surrounded by dotted-dashed lines are those including significant results (*p* < 0.05). The area within the cone of influence (continuous line) in the WPS indicates the region not influenced by edge effects.

The WPS of Sea Surface Temperature (SST) is displayed in Fig. 6a. As the global spectrum in the right panel shows, there is a large significance in the 4/5-year cycles. Fig. 6b displays the WPS of the cumulated average temperature, were a strong significant power at a 1-year scale (seasonal) is observed. The WPS of the precipitation is displayed in Fig. 6c, and it indicates a 1-year cycle as well as a 4–6-year cycle.

**Fig. 7:**
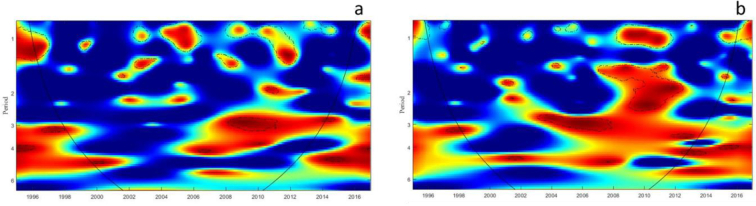
Wavelet coherence spectrum (WCS) of dengue incidence with (a) SST, (b) precipitation. The colors are coded as dark blue, for low coherence and dark red for high coherence between SST and dengue incidence time series. The y-axis of the WCS describe the periods in years (e.g., period 1: variables cohered at annual cycles); period 2,3,4: variables cohered at inter-annual cycles). The areas surrounded by dotted-dashed lines are those including significant results (*p* < 0.05). The cone of influence (continuous line) in the WCS indicates the region not influenced by edge effects.

The Wavelet Coherence Spectrum of dengue Cases and SST is displayed in Fig. 7a. There seems to be a small and intermittent relationship within the 1-year annual cycle, that likely reflects more localized or annual environmental factors that influence dengue transmission. The 3–5 year cycles show larger and more continuous areas of significance, indicating a stronger and possibly more consistent relationship between dengue cases and SST within the study period. Fig. 7b displays the coherence between dengue cases and Precipitation. The significantly coherent areas, which are outlined with the dashed line, appear to be on a 1-year cycle as well as at the 3 to 5-year cycles. The prominence of the 3–5 year cycle, which we observed to be more dominant, corresponds with the periodicity of La Niña events, part of the El Niño-Southern Oscillation (ENSO) cycle, which significantly influences climatic patterns across the globe, potentially influencing vector-borne disease dynamics like dengue fever.

**Fig. 8:**
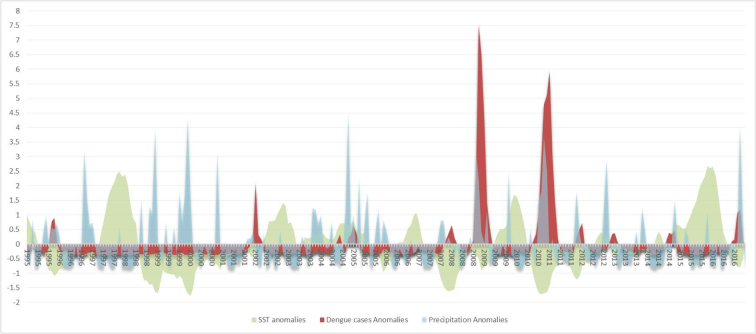
Standardized anomalies of Niño 3.4 (SST), precipitation and dengue cases in Curaçao.

The results displayed in Fig. 8 portray a pattern in which a decrease in SST (La Niña cooling phase) coincides with an increase in dengue cases and accumulated precipitation.

**Fig. 9:**
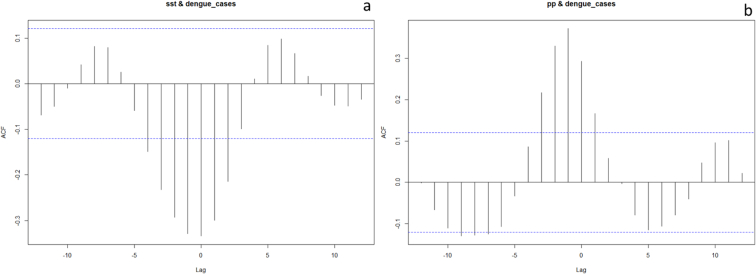
Cross correlation function of a) SST & dengue cases b) Precipitation & dengue cases.

The time series were further analyzed using cross correlation functions. The correlation between SST and dengue cases is presented in Fig. 9a. There is a statistically significant negative correlation between these variables, with a significant lag of up to four months. This implies that a lower SST results in more dengue cases in the following month and up to four months, albeit with a declining effect. The variables “precipitation” and “dengue cases” are correlated as well, although positively, which can be seen in Fig. 9b. This indicates that an increase in dengue cases can be explained by an increase in precipitation the previous month and up to three months prior.

## 4 Discussion

This study aimed to identify spatial as well as temporal trends in dengue virus infections on Curaçao between 1995 and 2016. Clusters of dengue infections were found throughout the study period and a significant link to population density has been established. Trends in the temporal dimension were found to be related to a combination of climatic variables which appear to be present on a multi-year cycle, clearly indicating a relationship with La Niña, the cooling phase of ENSO.

More specific results indicate that population density is linked to dengue transmission, and various analytical techniques were employed to identify hotspots of dengue infections. Additionally, a negative correlation was found between sea surface temperature and dengue cases in Curaçao, contrary to neighboring countries. Moreover, a positive correlation was identified between La Niña and dengue infections on the island, providing insights into the influence of ENSO cycles. This study's interdisciplinary approach contributes to better outbreak management by providing key information on specific hotspots and yearly and multi-year cycles of dengue for temporally and spatially targeted interventions.

### 4.1 Confirming spatial trends: population density and hot-spots

The findings from our spatial analysis provide valuable insights into the distribution and clustering of dengue cases in Curaçao. The observed spatial clustering of dengue cases in Curaçao is consistent with studies conducted in other regions. Sirisena et al. (Sirisena et al., 2017) investigated the spatial distribution of dengue cases in Sri Lanka and found significant clustering in urban areas with high population density. This pattern suggests that human movement and interaction play a critical role in dengue transmission dynamics. Exploratory results illustrate the population distribution and the density of dengue cases, with a clear concentration of cases around Willemstad, the capital city. The results from the mean and median center of outbreaks supported this observation, revealing that the mean and median center locations of dengue outbreaks were clustered and shifted over the densely populated northwest side of the island over time. Additionally, the assessment of spatial autocorrelation using Moran's I confirmed spatial autocorrelation throughout the dataset and for individual years. Furthermore, the hot spot analysis identified statistically significant hot spots in the vicinity of Schottegat, a densely populated area in the center of the island, as well as persistent hot spots in the western part of Curaçao. These findings suggest that the spatial distribution of dengue cases is not random, and is strongly influenced by variations in population density (Romeo-Aznar et al., 2022) and climatic factors.

### 4.2 Confirming temporal trends: local climate

Temporal variables such as climatic variables (precipitation and average temperature) are found to be determinants of dengue occurrence, given that these variables influence the life cycle and behavior of the Aedes mosquitoes, the primary vectors for dengue virus transmission (Kalra et al., 1997). This study identifies a relationship between a decrease in sea surface temperature and an increase in dengue cases in Curaçao, confirming the results of Limper et al. (Limper et al., 2016). Similarly, studies on Barbados reported that the risk of dengue is correlated with periods of excessive rainfall, mean monthly rainfall and the mean monthly relative humidity percentage (Lowe et al., 2018; Kumar et al., 2018).

Contrastingly, research in neighboring countries such as Venezuela and Colombia found the opposite effect, that an increase in temperature results in more dengue cases (Vincenti-Gonzalez, 2018; Cano-Pérez et al., 2022). This discrepancy may be explained due to the fact that Curaçao has a relatively high mean temperature compared to surrounding Caribbean countries. Temperatures below 30 °C have been found to be optimal for various aspects of the *Aedes aegypti* life cycle that are key for dengue virus transmission: flight performance (27 °C) (Rowley and Graham, 1968), reduced length of the gonotrophic cycle (26°- 30 °C) (Costa et al., 2010), and oviposition (26 °C) (Carrington et al., 2013). In accordance with this, the yearly average mean temperature on Curaçao fluctuates between 30 and 33 degrees °C, contrary to other countries in the Caribbean: Colombia (24 °C–27 °C), Venezuela (24–27 °C), Guyana (16 °C and 34 °C), Suriname (25 °C–27.5 °C) (World Bank, 2020). This inverse correlation between temperature and dengue infections has been further investigated in this study using cross correlation functions, which resulted in a negatively correlated lag effect between sea surface temperature and dengue infections as well as a positively correlated lag effect between precipitation and dengue infections. Lastly, it should be noted that the circulation of dengue serotypes in Curacao has not remained constant throughout the study period. The peaks of high incidence in the years 2001, 2008, and 2010 coincide with the years in which serotypes 1, 2, and/or 3 co-circulated on the island. This suggests a possible synergistic effect on the transmission or severity of the disease. The prevalence of different serotypes can have significant epidemiological implications. Co-circulation of serotypes can lead to dengue virus co-infections (Dhanoa et al., 2016), influence the pathogenicity of the infections, and alter the potential for severe disease outcomes (Martins et al., 2014). For example, secondary infections with a different serotype than the primary infection have been associated with an increased risk of severe dengue manifestations, such as dengue hemorrhagic fever (DHF) and dengue shock syndrome (DSS) (Guzman et al., 2013).

### 4.3 Novel insights: La Niña cooling phase related to an increase in dengue cases

This study provides novel insights into the relationship between dengue infections, sea surface temperature, and precipitation, particularly in the context of the El Niño-Southern Oscillation (ENSO) phenomenon. Our findings suggest that the impact of ENSO on dengue transmission extends beyond a yearly seasonality cycle. Instead, we identified significant associations between dengue outbreaks and longer cycles corresponding to ENSO phases, specifically highlighting the role of La Niña, the cooling phase of ENSO.

These findings diverge from previous research that mainly focused on the positive correlation between dengue outbreaks and El Niño, the warming phase of ENSO (Vincenti-Gonzalez et al., 2018; Xiao et al., 2017; Pramanik et al., 2020). The contrast in our results underscores the complexity of the relationship between ENSO and dengue infections, indicating that different ENSO phases may have varying effects on dengue transmission dynamics. This distinction may be attributed to regional variations, local climate conditions, and vector abundance, which can modify the impact of ENSO on disease transmission in different geographical areas.

Understanding the influence of La Niña on dengue outbreaks in Curaçao holds significant implications for public health preparedness and response. La Niña events can be predicted up to six months in advance, providing a valuable opportunity for local authorities to anticipate and proactively address potential dengue outbreaks. By leveraging climate forecasts and ENSO predictions, public health officials can implement targeted interventions and resource allocation strategies to effectively mitigate the impact of dengue infections during La Niña events. However, further research is necessary to comprehensively elucidate the underlying mechanisms that link La Niña with increased dengue cases. Factors such as altered mosquito behavior, changes in breeding sites, and shifts in human behavior during La Niña conditions may play a role in facilitating dengue transmission. Moreover, the interaction between climatic drivers, socioeconomic factors, and public health practices may be complex and multifaceted, contributing to the observed associations.

### 4.4 Value of multiple analysis methods

An important aspect of this study is the use of various analysis techniques. The different methods, such as the mean and median center, hot spot analyses and temporal analyses, have been fundamental to our understanding of the spatial and temporal trends of dengue virus infections on Curaçao. It is the combination of these various analysis techniques that strengthen the results of this study. The two different hot spot analysis techniques identified similar hotspots, increasing their validity. The combination of wavelet analysis with cross correlation functions provided insights into different aspects of the effect of climate variables on dengue cases, such as yearly seasonal trends, as well as the effects of 4- or 5-year cycles.

### 4.5 Contribution to practice

This study aimed to contribute to a more effective dengue outbreak management. The spatial analysis identified multiple hot spots of dengue cases, which allows local authorities to adequately allocate funds to target specific hot spots of disease occurrence. Additionally, the temporal analysis brought insights into the seasonality of dengue cases on a yearly basis, which allows local authorities to develop a strategy for temporal targeting. Finally, the identified relationship between dengue cases and La Niña is relevant here, as the occurrence of La Niña can be predicted up to six months prior to the event, which makes it possible for local authorities to more effectively predict and target dengue hotspots in advance. The combination of spatial and temporal analysis provided insights that can improve the targeting of dengue management, both in space and time.

## 5 Limitations

The findings in this research are subject to at least three limitations. Firstly, the geographical data has gone through many stages of editing in the geocoding process, which may have decreased the accuracy of the location of dengue cases data. Secondly, the cases were analyzed at household level, which do not necessarily reflect the location of infection. Finally, only recorded cases are included in this study, while non-registered cases are most likely present but not included. Even though these data were the best available option, the spatial data trends might have been affected by biases resulting from unbalanced recording of cases across space.

## 6 Conclusion

The findings of this study are consistent with those of prior investigations that have explored the impact of population density on dengue transmission (Sirisena et al., 2017; Vincenti-Gonzalez, 2018; Elsinga, 2018). Dengue is a multifaceted vector-borne illness that is heavily influenced by geographical factors, including human movement, mosquito density, and population density. Throughout this investigation, various analytical techniques were employed to identify hot spots and seasonality (yearly and multiyear) of dengue infections, revealing locations with a significantly elevated incidence of the disease. This study has shown that a multi-analytical approach is useful and necessary to understand the complex spatial dynamics of dengue outbreaks, and may also be applied for the surveillance of various communicable diseases. Such an approach can assist regulatory decision-making and optimize resource management for outbreak response in the affected region by better preparing outbreak management on both the spatial level, by targeting specific hot spots, as well as on the temporal level, by utilizing the knowledge on yearly and multiple-year cycles. The results of this study are expected to have similar implications for islands in proximity to Curaçao, such as Aruba and Bonaire, due to the similar climatic and geographical profiles of these islands.

Future studies can build upon this research in two different ways. Firstly, within Curaçao, by further investigating the identified dengue clusters, as well as by including other variables such as socio-economic, public services availability and frequency, and dengue-related preventive measures variables. Secondly, outside of Curaçao, the relationship between La Niña and dengue cases may be further investigated. There are specific geographical and climate related variables which create the circumstances in which the mosquitos carrying the dengue virus appear to thrive. It is important to study the trends and patterns of dengue infections at a local level, taking into consideration the climatic and country-specific characteristics to draw more accurate assumptions for epidemic preparedness.

## Declaration of competing interest

The authors declare no competing interests.

## Figures and Tables

**Figure:**
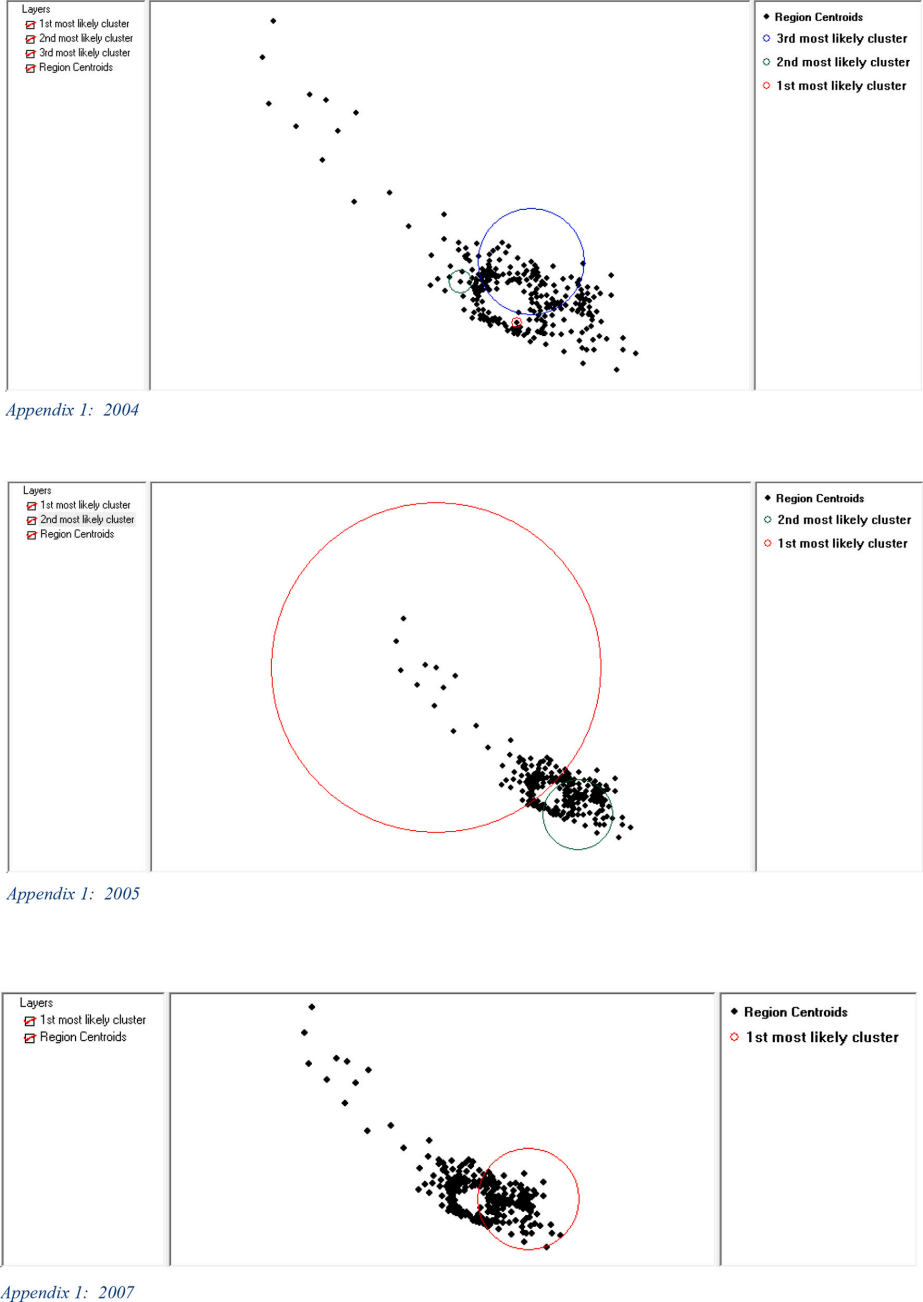

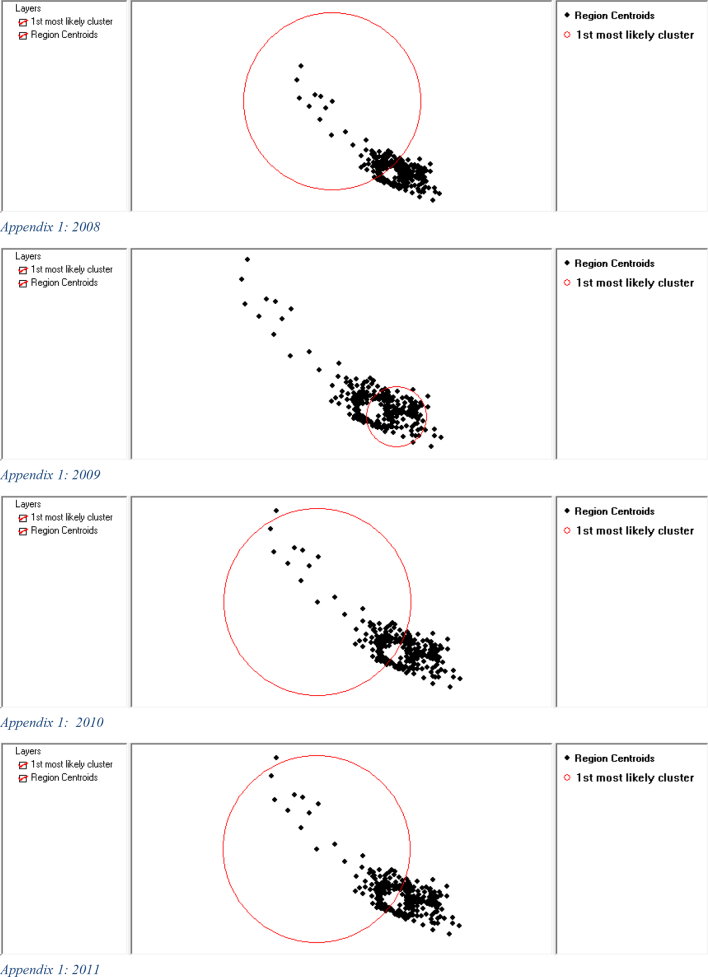

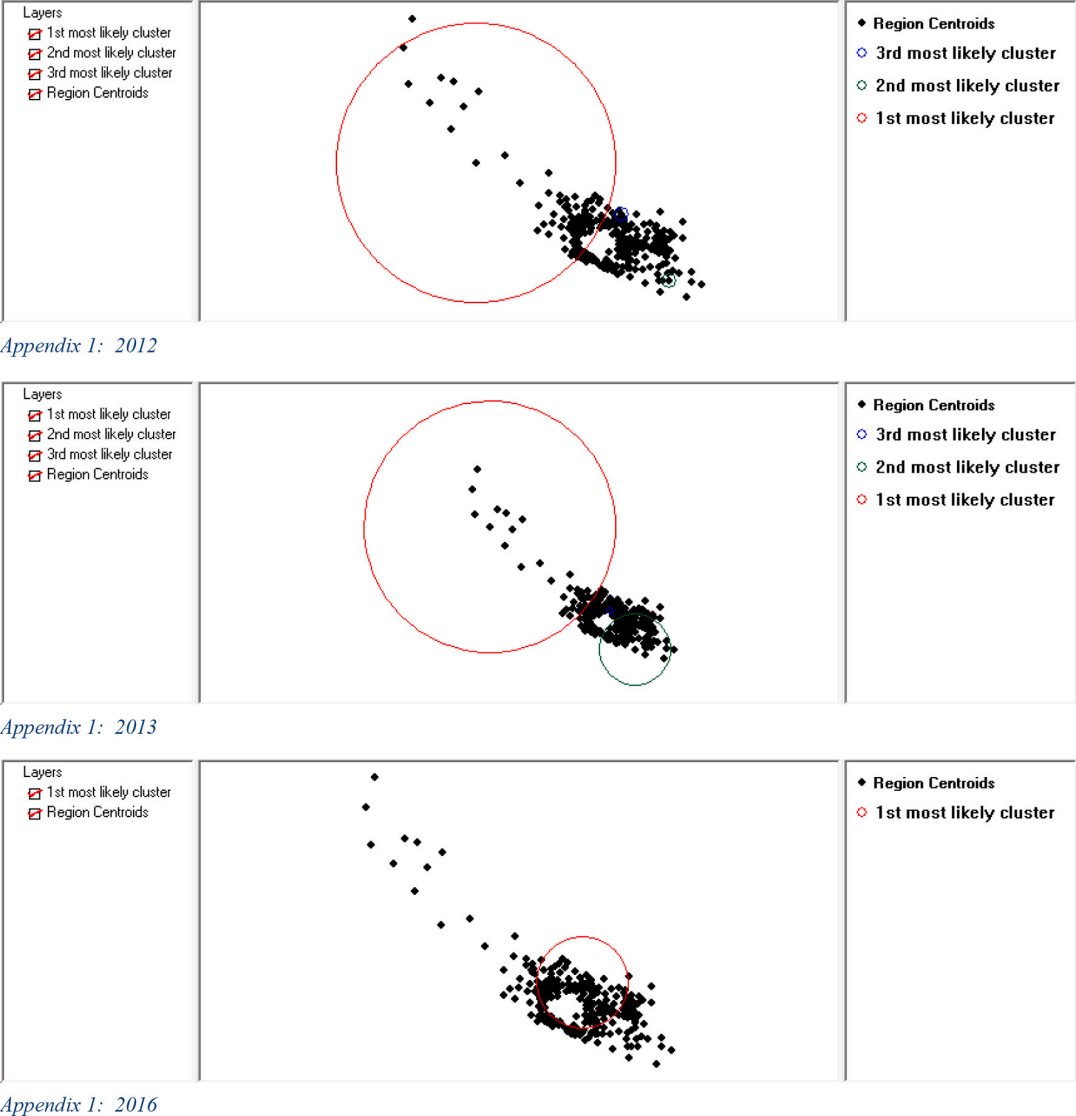


**Figure:**
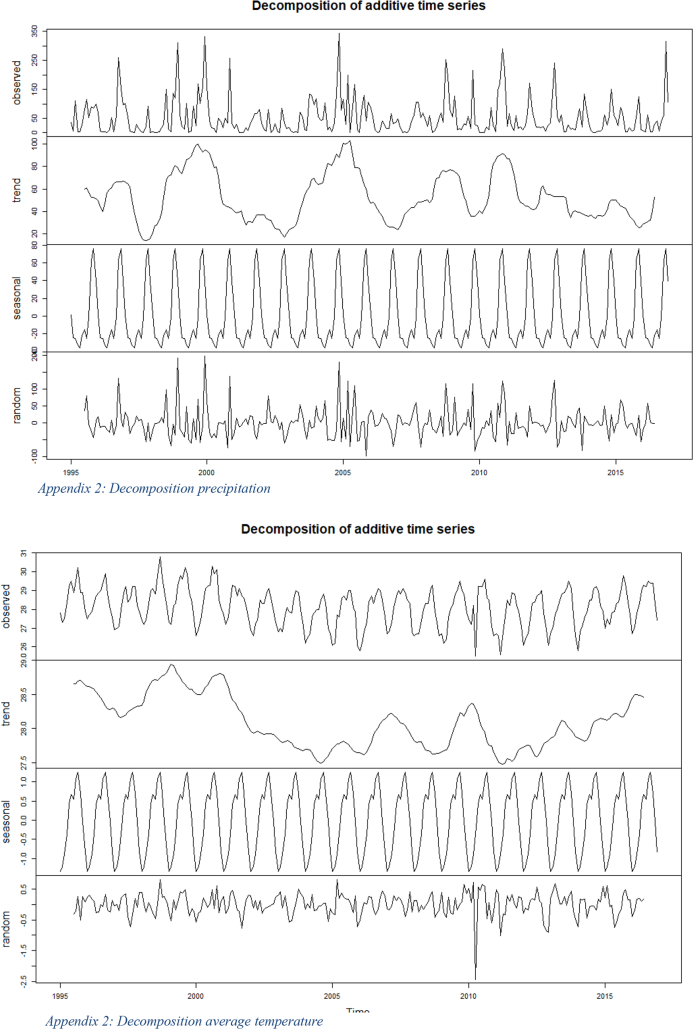


## Data Availability

Climatic data for Curaçao is available on: https://www.meteo.cw/. Sea surface temperature data is available from the Climate Prediction Center of the National Oceanic and Atmospheric Administration (CPC) on: https://www.cpc.ncep.noaa.gov/data/indices/ Data on dengue infections is not publicly available due to privacy regulations. Dengue infections data can be requested at the Curaçao Ministry of Health.
